# Protective role of trehalose during radiation and heavy metal stress in *Aureobasidium subglaciale* F134

**DOI:** 10.1038/s41598-017-15489-0

**Published:** 2017-12-14

**Authors:** Tingting Liu, Liying Zhu, Zhiping Zhang, He Huang, Zhidong Zhang, Ling Jiang

**Affiliations:** 1 College of Food Science and Light Industry, Nanjing Tech University, Nanjing, 210009 People’s Republic of China; 2 College of Biotechnology and Pharmaceutical Engineering, Nanjing Tech University, Nanjing, 210009 People’s Republic of China; 3 College of Chemical and Molecular Engineering, Nanjing Tech University, Nanjing, 210009 People’s Republic of China; 4 Nanjing Beishengrong Energy Technology Co. Ltd, Nanjing, 210009 People’s Republic of China; 5 Institute of Microbiology, Xinjiang Academy of Agricultural Sciences, Urumqi, Xinjiang Uigur Autonomous Region People’s Republic of China

## Abstract

An isolated black yeast-like strain was obtained from radiation-polluted soil collected from Xinjiang province in northwest China. On the basis of ITS and LSU rDNA sequence analysis, in combination with the colony morphology and phenotypic properties, the isolated strain was revealed to represent a novel variety of *Aureobasidium subglaciale*, designated as *A. subglaciale* F134. Compared to other yeasts and bacteria, this isolate displayed superior resistance to gamma irradiation, UV light, and heavy metal ions. It was discovered that the resistance of the isolate was correlated with the stress protector trehalose. Through the overexpression of the trehalose-6-phosphate synthase gene *tps1* and the deletion of acid trehalase gene *ath1*, the AP*T∆A* double mutant exhibited a survival rate of 1% under 20 kGy of gamma-radiation, 2% survival rate at a UV dosage of 250 J/m^2^, and tolerance towards Pb^2+^ as high as 1500 mg/L, which was in agreement with the high accumulation of intracellular trehalose compared to the wild-type strain. Finally, the protective effects and the mechanism of trehalose accumulation in *A. subglaciale* F134 were investigated, revealing a significant activation of the expression of many of the stress tolerance genes, offering new perspectives on the adaptations of radioresistant microorganisms.

## Introduction

The production and testing of nuclear weapons will release radioactive waste into the environment, which are now contaminating the ground and subsurface at thousands of sites. In China, the largest nuclear test to date was conducted on 21 May 1992 in the Kuruktag and Kyzyltag mountains of Xinjiang Uyghur Autonomous Region, with pollution covering large areas of the surface and underground. These highly toxic waste sites become the ideal places to search for radiation resistant microorganisms^1^. Recently, various radiation resistant organisms have been isolated by our group from the radiation-contaminated soils in the Xinjiang Uyghur Autonomous Region of Northwest China, belonging to the domains bacteria, archaea and fungi, and several of their stress-resistance genes have been identified by genome sequencing^2–7^. In the existing nuclear pollution areas, such as the Chernobyl Atomic Energy Station (ChAES), 37 species belonging to 19 genera in the eucarya domain had been successfully discovered before 2000^8^, which was expanded into 200 species from 98 genera in 2004^9^. However, there is an even richer biodiversity at the Xinjiang nuclear explosion area due to adaptation and evolution for more than 50 years^10^.

Trehalose is a naturally stable, non-reducing disaccharide sugar, in which two glucose units are linked via an α,α-(1,1)-glycosidic bond^11^. It has been isolated from a large number of prokaryotic and eukaryotic species spanning bacteria, archaea, yeast, fungi, algae, plant, as well as low orders of the animal kingdom, especially those living in extreme environments^12^. So far, five different enzymatic pathways related to the biosynthesis of trehalose have been uncovered and identified in naturally occurring microorganisms^13^. Yeast-like cells, mycelia and melanin-pigmented chlamydospores of genus *Aureobasidium* are particularly known for their biotechnological significance, since this organism is a producer of the extracellular polysaccharide (EPS) pullulan (poly-α-1, 6-maltotriose)^14–16^. As the type species, *Aureobasidium pullulans* is an ubiquitous oligotroph that can be found in various environments such as hypersaline waters in salterns^17^, on rocks and monuments^18^, as airborne spores^19^, in food and feeds^20^, in deserts^21^, and in industrial effluents^22^. As previously reported, one copy of the trehalose biosynthesis gene encoding trehalose-6-phosphate synthase (TPS) was found in four investigated *A. pullulans* varieties, and another relatively dissimilar trehalose-related hydrolase (e.g., trehalase, EC 3.2.1.28) was also identified, which hydrolyzes trehalose into glucose^15,23^. In addition, the concentration of trehalose in *A. pullulans* increased when the cells were exposed to high salt concentrations or high temperatures^24^. However, the biological effects and protective mechanisms of trehalose accumulation in *A. pullulans* cells exposed to irradiation and heavy metal stress remain unknown.

In this research, a new black yeast-like fungus was isolated from radiation and heavy metal-polluted soil samples from Xinjiang, Uyghur Autonomous Region of Northwest China. Based on ITS and LSU rDNA sequence analysis, the isolated strain belongs to the genus *Aureobasidium* and represents a novel isolate of *A*. *subglaciale*. Furthermore, we generated a trehalose-overproducing mutant strain of *A*. *subglaciale* F134 by overexpressing *tps1* and disrupting *ath1*. The mutant strain showed a 3-fold increase in biosynthesis of trehalose, and furthermore displayed enhanced resistance characteristics, confirming that there is an interplay between trehalose metabolism and the oxidative stress response in *Aureobasidium*.

## Results and Discussion

### Strain isolation and phylogenetic analyses

**Figure 1:**
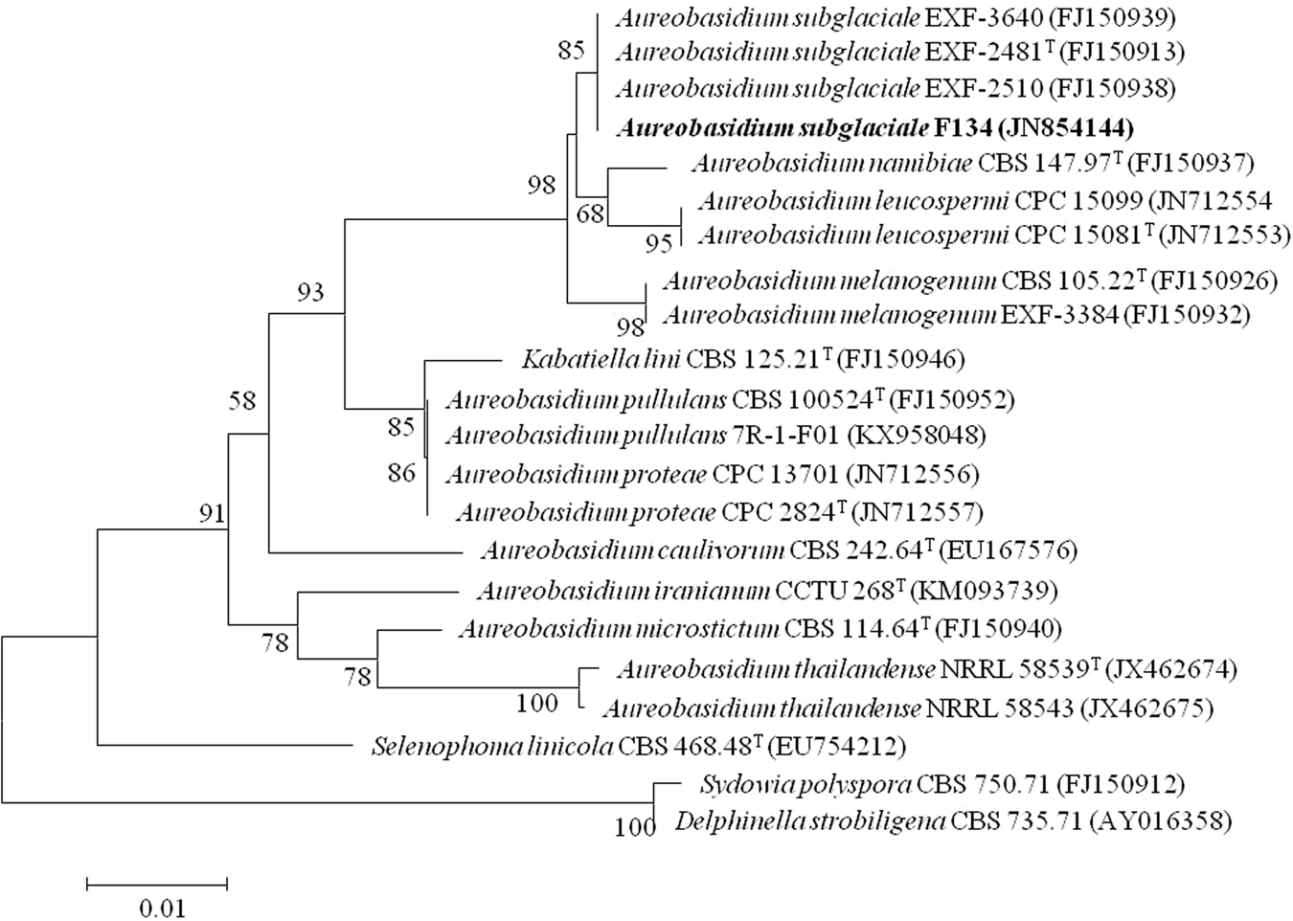
Phylogenetic tree, reconstructed based on the D1/D2 domain of LSU rRNA gene by using the neighbor-joining method implemented with MEGA version 7.0, showing that the strain *A. subglaciale* F134 had a great similarity with relevant species in the geuns *Aureobasidium*. Bootstrap values >50% were shown on the branches and bootstrap values were set at 1000 iterations. The scale bar was considered as an evolutionary distance of 0.01 nucleotide substitution rates.

A total of 13 isolates comprising 6 strains of mycelial fungi and 7 strains of yeast from the polluted soil samples were obtained on Czapek^25^ and potato dextrose agar (PDA)^26^ plates containing different concentration of Pb^2+^. As a result, only one isolated yeast-like strain grew well in the presence of 0.12% Pb^2+^ on PDA plates, and the morphological analysis indicated that this isolate was highly likely to be a member of the genus *Aureobasidium* (Figures S1, S2)^27^. According to sequence comparisons of the internal transcribed spacer (ITS) region and the D1/D2 domains of the large-subunit (LSU) rRNA gene, the isolated strain was preliminarily identified as a novel yeast strain of the genus *Aureobasidium*. A phylogenetic tree study was further performed with the LSU rRNA gene sequences of type strains of all the species from the genus *Aureobasidium* (Fig. 1). According to the similarity matrix based on the alignment of all cited representative LSU rRNA gene sequences, a high level of support indicated that the strain was closely related to *A*. *subglaciale*, which is member of the secondary clade and shared a 85% homology with *A. subglaciale* EXF-2510 (FJ150938), *A. subglaciale* EXF-2481T (FJ150913) and *A. subglaciale* EXF-3640 (FJ150939). Based on above results, we proposed to name this isolated strain of *Aureobasidium* as *A*. *subglaciale* F134, which could be readily distinguished from the type strain of *A. pullulans* ^16^ by a combination of phenotypic properties (Table S1).

### Trehalose metabolism in *A. pullulans*

The most widespread trehalose pathway is OtsA-OtsB, which is found in all the prokaryotic and eukaryotic organisms^12^. This pathway starts when trehalose-6-phosphate synthase (TPS) catalyzes the transfer of glucose from UDP-glucose to glucose-6-phosphate (G-6-P) with the formation of trehalose-6-phosphate (T-6-P) and uridine diphosphate (UDP). The T-6-P is therewith dephosphorylated into trehalose by trehalose-6-phosphate phosphatase (TPP). In additon, trehalose can be hydrolyzed to glucose by either a cytosolic neutral trehalases (NTH) or a vacuolar acidic trehalase (ATH), which may bring the levels of trehalose back to normal equilibrium levels once the stress is over^28^. Previous studies have successfully engineered yeast strains through the overexpression or deletion of trehalose metabolic genes (*tps1*, *tps2*, *tps3*, *tsl1*, *ath1*, *nth1*, and *nth2*), with the aim to investigate the protective function of trehalose under various stress conditions^28,29^. Importantly, the availability of genome sequence data for *A. pullulans* EXF-150 and *A. pullulans* AY4 made it possible to screen the new isolate from this study for the presence of ORFs sharing homology with genes related to trehalose metabolism^16,30^. The ORFs with high similarity to apparent orthologs from three model microorganisms involved in the synthesis and degradation of trehalose were found in *A*. *subglaciale* F134 and the results are summarized in Table 1. As can be seen in Table 1, a BLAST search mainly based on the known *Saccharomyces cerevisiae* pathway genes identified ORFs with putative functions as a homologue of Tps1 and Ath1 as having 69% and 32% overall identity respectively, at the amino acid level. As a result, the most highly expressed genes *tps1* and *ath1* were chosen for engineering by overexpression and deletion, respectively, with the aim to improve intercellular trehalose accumulation.

**Table 1:** Homologues of trehalose biosynthesis enzymes and their identity in *A*. *subglaciale* F134.

		Matching sequences			
*A. subglaciale ORFs*	Length (aa)	strain	Description	Length (aa)	Identity (%)
Tps1 (putative)	541	*Saccharomyces cerevisiae*	trehalose 6-phosphate synthase Tps1	495	69%
		*Arabidopsis thaliana*	trehalose 6-phosphate synthase Tps1	942	49%
		*Escherichia coli*	cytoplasmic trehalase	549	36%
Ath1 (putative)	792	*Saccharomyces cerevisiae*	alpha, alpha- trehalase ath1	1211	32%
		*Arabidopsis thaliana*	vacuolar acid trehalase	473	31%
		*Escherichia coli*	trehalose-6-P hydrolase	551	78%

**Figure 2:**
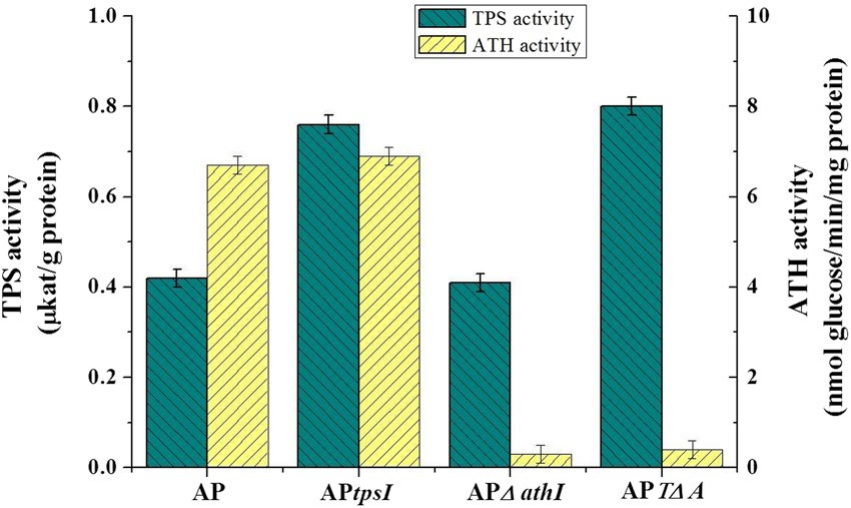
The enzyme activities of TPS and ATH between wild strain AP and its engineered strains AP*tps1*, AP*∆ath1* and AP*T∆A* were compared. Each error bar indicates +/− one standard error of the mean.

In order to demonstrate the effects of genetic manipulation, the enzyme activities of TPS and ATH as well as the intercellular trehalose contents of the wild-type strain AP and its engineered derivatives were compared. As expected, the engineered strain AP*tps1* had higher TPS activity, strain AP*∆ath1* had lower ATH activity, and strain AP*T∆A* had a higher TPS and a lower ATH activity, compared with the wild-type strain (Fig. 2). Therefore, the three engineered strains all showed a significant increase of intracellular trehalose levels compared with their wild-type parent strain (Table 2). Among these engineered strains, the strain AP*T∆A* exhibited a 3-fold increase in trehalose content without stress induction (39.3 ± 1.9 vs. 13.1 ± 0.6 mg trehalose/g dry cell weight) and even more in the presence of Pb^2+^ (212.3 ± 10.8 vs. 62.1 ± 3.1 mg trehalose/g dry cell weight), which demonstrated the significant effect on trehalose content that can be achieved by the overexpression of TPS1 combined with deletion of ATH1 in *A. pullulans*.

**Table 2:** Intracellular trehalose levels of *A. subglaciale* F134 wild-type and trehalose biosynthesis mutants under different conditions^a^.

Strains	Trehalose yield (mg/g dry weight)	
	YPD	Pb^2+^ (1,000 mg/L) with YPD
AP	13.1 ± 0.55	62.1 ± 3.12
AP*tps1*	25.8 ± 2.24	110.7 ± 5.49
AP*∆ath1*	19.5 ± 0.99	98.2 ± 3.75
APT*∆A*	39.3 ± 1.87	212.3 ± 10.8
P^b^	0.00	0.00

^a^Strains were precultured in YPD medium to stationary-phase, and cells were collected and cultured in YPD with or without the stress factors at 28 °C with 200 rpm for 10 h. All data shown are mean values from at least three replicate experiments.

^b^P < 0.05 indicates there are statistically significant differences between the four strains.

### Resistance to radiation and heavy metals

Stress tolerance is often associated with accumulation of small organic molecules, which can act as protective compounds against various stress factors^31^. In yeasts and other microorganisms, a large body of research suggests that trehalose serves not only as carbohydrate storage molecule, but also as a metabolic regulator and protective agent against a wide variety of abiotic stresses, such as heat^32^, cold^33^, dehydration^34^, desiccation^35^, and oxygen radicals^36^. Therefore, we examined the growth characteristics of the wild-type AP and the mutant strain AP*T∆A* under radiation and heavy-metal stress conditions.

**Figure 3:**
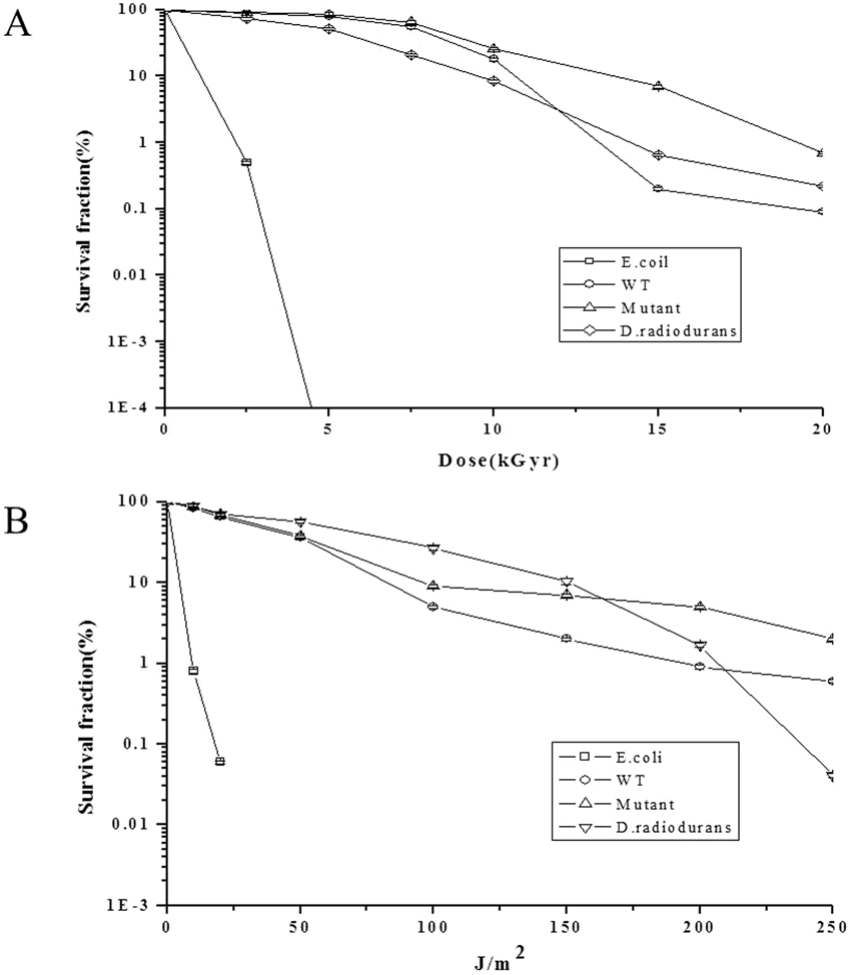
Survival fraction of the isolates exposed to gamma and UV rays. (**A**) Doses of gamma rays. (**B**) Doses of UV rays. The isolates of *Aureobasidium* strain wild-type AP and modified mutant strain AP*T∆A* were test with *E. coli* as a negative contrast and *D. radiodurans* as a positive contrast. Each error bar indicates the average value obtained in the three experiments.

Gamma-radiation and UV-light survival curves for both the wild-type and the mutant strain AP*T∆A* showed a characteristic shoulder form, as opposed to the nearly linear inactivation kinetics observed for *Escherichia coli* (Fig. 3). As can be seen in Fig. 3, there was a decrease in the number of CFU/g recovered from the wild-type AP strain with increasing doses of gamma rays and increasing irradiation time with UV light. Significantly, this lethality trend was slowed significantly in the mutant strain AP*T∆A*. For example, after exposure to 20 kGy, less than 0.1% of the original culturable AP cells could be recovered by dilution plating, while the surviving fraction was 10-times larger, at nearly 1%, in the mutant strain AP*T∆A*. Even so, the genus *Aureobasidium* is still resistant to levels of gamma radiation that far exceed the background levels in the natural environment, and was obviously superior to *Phaffia rhodozym*a with a survival rate of 0.1% under a lower dosage of 3 kGy^37^, and *Saccharomyces cerevisiae* with a survival rate of less than 0.01% under 150 J/m^2^ ^38^. Moreover, compared with the well-known radiation-resistant bacterium *Deinococcus radiodurans* R1, the survival rates of both the wild-type and the mutant strain AP*T∆A* were in the equivalent order of magnitude when exposed to the same does of gamma and UV radiation, including an extraordinary performance at UV does as high as 250 J/m^2^ (Fig. 3), which makes them compare favorably with the world’s most recognized radioresistant microorganisms^39^.

**Figure 4:**
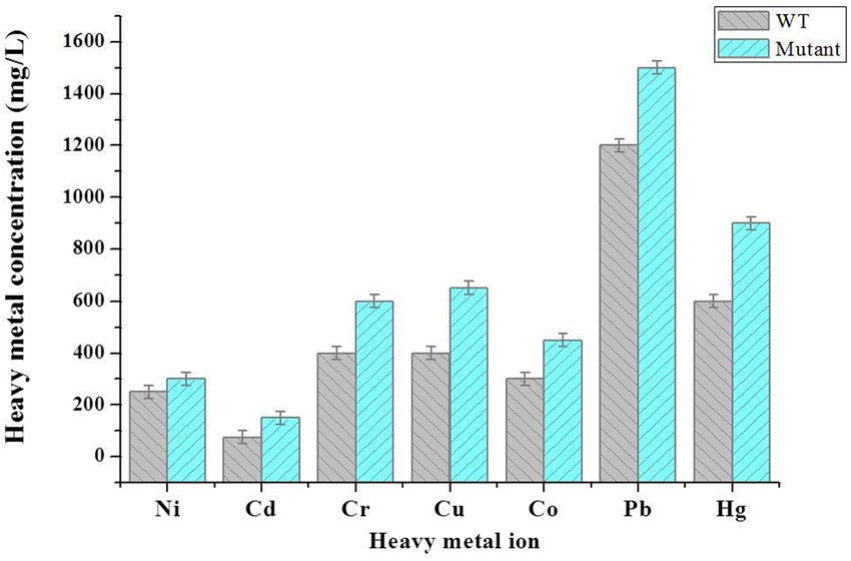
The resistance to different concentrations of heavy metal of isolates of *Aureobasidium* strain wild-type AP and modified mutant strain AP*T∆A*. Each error bar indicates +/− one standard error of the mean.

Additionally, the resistance of both the wild-type AP and the mutant strain AP*T∆A* to different heavy metals was also investigated (Fig. 4). Members of the genus *Aureob* *as* *idium* showed different degrees of tolerance to different heavy metals, with MTCs for heavy metals ranging from 75 to 1,500 mg/L. The resistance test indicated that the AP strain exhibited a very high degree of resistance to almost all of the investigated heavy metals, and especially to Pb^2+^, to which it showed more than twice higher tolerance than to the others. It is well-known that heavy metals are toxic to living cells even at low concentration of about 1.0–10 mg/L, and some, such as Hg and Cd, are very toxic even at lower concentration of 0.001–0.1 mg/L^40^. The heavy metal tolerance test indicated maximum microbial tolerance of *Pseudomonas* sp. to Cu at 300 mg/L^41^, and *Streptomyces* sp. to Hg at 200 mg/L^42^, while 100 mg/L Cd was considered as the MTC of the metal for most of the fungus^43^. Furthermore, we have shown that the ability of cells to withstand the lethal effect of heavy metals rises with the increase in their trehalose content. The AP*T∆A* double mutant strain was relatively more tolerant to most heavy metals under investigation, with MTC increases ranging from 20 to 60%. Overall, the pattern of metal tolerance was in the order of Pb^2+^ > Hg^2+^ > Cu^2+^ > Cd^2+^ > Co^2+^ > Ni^2+^ > Zn^2+^.

In order to investigate the roles of relevant genes in the alteration of the cellular phenotype in relation to heavy metal stress tolerance between the wild-type AP and the mutant strain AP*T∆A*, qRT-PCR was performed. Genes involved in DNA repair (e.g., *rad1*, *rad25*, *mutL*) and the partial oxidases, such as *FeSOD*, *ccp1*, *caos* and *gsho1*, displayed strikingly increased expression levels in the mutant strain AP*T∆A* (>10-fold). Moreover, genes involved in oxidative stress (e.g., *svf1*, *rci1*) and transporter genes (e.g., *ABC1*, *qutD*) were up-regulated 2–4 fold when trehalose was overproduced in the mutant cells (Table 3). Trehalose is a stress-induced metabolite and has the ability to protect the cells as a whole, as well as the cell membranes, proteins and other biological macromolecules under various adverse environmental conditions^35^. In this study, the strongest evidence for the protective role of trehalose was that the mutant strain AP*T∆A* with more trehalose accumulation had a markedly enhanced resistance to Pb^2+^. Most likely, trehalose enhances resistance to heavy metal stress by quenching cations generated either in the medium or inside cells after entry of metal ions (e.g., Pb^2+^), through the promotion of the expression of the above functional genes. This observation was consistent with a previous report on cells exposed to a free radical-generating system (H_2_O_2_/iron)^36^. Our previous study demonstrated that trehalose also improved the robustness of *Propionibacterium acidipropionici*, especially in response to acid stress^44^. However, this is, to the best of our knowledge, the first study to directly demonstrate that the ability of microorganisms to survive under heavy metal stress is related to the intracellular concentration of trehalose.

**Table 3:** qRT-PCR results of genes related to Pb^2+^ tolerance^a^.

Gene		Expression level (Avg. ± std)		Fold change (APT*∆A*/AP)
Abbr.	Full name of expression product	Wild-type AP	Mutant APT*∆A*	
*rad25*	DNA repair helicase	2.12 ± 0.45	38.48 ± 1.97	18.15
*rad1*	DNA repair exonuclease	3.66 ± 1.01	62.66 ± 3.32	17.12
*mutL*	DNA mismatch repair protein	1.98 ± 0.87	30.43 ± 1.56	15.37
*svf1*	Oxidative stress survival protein	13.28 ± 1.64	29.61 ± 1.86	2.23
*rci1*	Oxidative stress response RCI peptide	17.83 ± 0.89	56.88 ± 2.99	3.19
*ABC1*	ATP-binding cassette transporter	21.01 ± 1.67	60.51 ± 3.23	2.88
*qutD*	Major facilitator superfamily (MFS) transporter	10.16 ± 2.18	37.90 ± 2.37	3.73
*FeSOD*	Iron superoxide dismutase	4.11 ± 1.28	47.51 ± 2.41	11.56
*ccp1*	Cytochrome c peroxidase	2.09 ± 0.33	22.95 ± 1.24	10.98
*caos*	Copper-containing amine oxidases	1.55 ± 0.19	18.80 ± 1.12	12.13
*gsho1*	Glutathione peroxidase	3.39 ± 1.16	54.69 ± 2.74	16.13

^a^The concentration of lead ion (Pb2+) was 0.10% (1,000 mg/L). All data are obtained as the mean of three tests with P < 0.05.

## Conclusion

Samples from soils polluted by radiation and heavy metals were collected in Xinjiang, Uyghur Autonomous Region of Northwest China, and a novel isolate was selected on enrichment media containing Pb^2+^. The phylogenetic tree based on the LSU rRNA gene suggested that this isolate should be classified as a novel strain of *Aureobasidium subglaciale* and was therefore designated as *A. subglaciale* F134. In addition, the trehalose content of strain F134 has been manipulated through the overexpression of the trehalose-6-phosphate synthase gene *tps1* and deletion of the acid trehalase gene *ath1*. Compared with the wild-type AP strain, the mutant strain AP*T∆A* had a 3-fold increased trehalose content, which significantly improved both its radiation- and its heavy-metal tolerance. Further investigation of the relevant genes related to heavy metal stress tolerance showed that genes encoding DNA repair proteins, oxidases, oxidative stress response factors, and transporter proteins were up-regulated dramatically when trehalose was overproduced in the mutant cells. This study is the first to report on the protective effects of trehalose against radiation and heavy metal stress in the genus *Aureobasidium*, and expands our knowledge of the diversity of radiation and heavy metal-resistant microorganisms.

## Materials and Methods

### Sampling sites and sample collection

Samples were taken from Chinese nuclear radiation polluted areas located in the Xinjiang Uyghur Autonomous Region of Northwest China. The samples were collected in October 2011 from the middle level of pollution soils (1,000–2,000 Bq/kg)^45^ at a depth of approximately 5–20 cm, and were immediately preserved in sterile centrifuge tubes (50 mL) at 4 °C, and transported to the laboratory in dark conditions.

### Selective media and isolation

Approximately 2.0 g of each sample was added to the liquid enrichment media as inoculum. The enrichment media were as follows: Czapek medium, PDA medium, MEA medium, and YPD medium. All media were supplemented with chloramphenicol (50 mg/L) to selectively inhibit the growth of bacteria.

The soil samples were filtered through a 2 mm pore size mesh, and approximately 1–2 g portions were individually transferred into 15 × 150 mm aseptic tubes with 5 mL of sterile Czapek liquid medium and cultivated under shaking at 28 °C for 3 days. The resulting enrichment cultures were diluted 10, 20 and 30-fold, and 0.02% of the dilution was spread on plates comprising Czapek or PDA agar, both with 0.05% Pb^2+^, and incubated at 28 °C for 7 days. Colony growth was observed daily, and single colonies were selected based on colony characteristics, transferred to fresh agar plates for further purification and stored on PDA agar slants at 4 °C for later identification and characterization.

### Molecular identification

Fresh yeast cells were transferred into MEA broth and cultured under shaking at 28 °C for 60 h. The cells were collected by centrifugation and resuspended in TE buffer 3 times, after which the genomic DNA was extracted using the E.Z.N.A.^TM^ Yeast DNA Spin Protocol Kit (Omega Bio-Tek, Georgia, USA) according to the manufacturer’s instructions, and stored at −20 °C for further study. The sequences of the ITS region and the D1/D2 region of the LSU rRNA gene were amplified from the extracted *A. pullulans* genomic DNA using the primers ITS1 (5′-TCCGTAGGTGAACCTGCGG-3′), ITS4 (5′-TCCTCCGCTTATTGATATGC-3′), and the primers NL-1 (5′-GCATATCAATAAGCGGAGGAAAAG-3′), NL-4 (5′-GTCCGTGTTTCAAGACGG-3′), respectively. The polymerase chain reaction (PCR) was carried out using the following amplification conditions: 95 °C for 5 min, followed by 35 cycles comprising 94 °C for 1 min, 52 °C for 1 min and 72 °C for 2 min, followed by a final elongation step at 72 °C for 10 min. Before sequencing, the amplified PCR products were purified using the Axy Prep^TM^ DNA Gel Extraction Kit (Qiagen, Chatsworth, CA), and the concentrations of the purified products estimated based on band intensity following agarose gel electrophoresis. DNA was sequenced using the ABI3730 sequencer (Applied Biosystems, Foster City, CA) with the indicated primers, to obtain a result permitting an identification of the taxonomic status of the strain.

The sequence data was integrated from bidirectional sequencing using software ContigExpress (Vector NTI Suite 6.0, InforMax Inc.). BLAST was used to pairwise compare the obtained sequences with information of related species from the GenBank. MEGA (version 7.0) was used to reconstruct a phylogenetic tree and conduct cluster analysis by using the neighbor-joining method, and ascertain the biological classification status of the isolated strain. The D1/D2 domain of the LSU rRNA gene was aligned automatically using ClustalW. The generated phylogeny was tested using the bootstrap method set at 1000 iterations. Bootstrap analysis was used to estimate the confidence levels of the clades, and values that were greater than 50% were displayed on the phylogenetic tree.

### Genetic manipulation of the trehalose biosynthesis pathway

To overexpress the TPS1 gene, the corresponding DNA fragment was amplified by PCR from the chromosomal DNA of *A. pullulans* and ligated into the *Kpn*I-*Apa*I digested plasmid pUG6E, containing the kanMX resistance marker. The TPS1-overexpression strain was obtained through the homologous recombination of the *Stu*I-linearized plasmid pUG6E-TPS1 and the TPS1 gene in the genome. The transformants were selected on YPD plates containing 300 μg/mL G418. The engineered haploid was named AP*tps1* (MAT α, PGK-TPS1-kanMX).

Gene disruption using the disruption cassette was performed based on the method by Güldener *et al*.^46^. The *ath1* deletion strain was obtained by a one-step disruption of ATH1. The ATH1 disruption cassette contained, from left to right, the ATH1A fragment comprising the 500 bp upstream of the ATG start codon of ATH1, the *ble* gene, and the ATH1B fragment comprising the 500 bp downstream of ATH1. The strains AP and AP*tps1* were transformed with the ATH1 deletion cassette, and the ath1 knockout strains were selected on YPD medium containing 50 μg/mL zeocin. The *ath1* knockout strain was named AP*∆ath1* (MAT α, *∆ath1*::*ble*), and the mutant strain with *tps1* overexpression and *ath1* deletion was named APT*∆A* (MAT α, PGK-TPS1-kanMX, *∆ath1*::*ble*).

The *A. subglaciale* strains were transformed using the LiAc/single-stranded carrier DNA/PEG method^47^. Proper integration was verified via diagnostic PCR and sequence analysis.

### Radiation resistance analysis

Cell suspensions (OD_600_ ~1.0) were divided into 2 mL aliquots and exposed to a gamma source at a dose rate of 100 Gy/min at room temperature; the delivered gamma radiation doses spanned from 0 to 15.0 kGy in steps of 2.5 kGy. The thus treated samples were spread directly or after suitable serial dilution onto MEA agar plates and incubated in the dark at 28 °C for 10 days to count the colony-forming units.

UV irradiation experiments were performed using a 15 W UV-B lamp. Cell suspensions (OD_600_ ~1.0) were divided into 10 mL aliquots and placed into 9 cm diameter plates. The plates were exposed to UV light at a distance of 30 cm. The time of UV irradiation was from zero to 15 min in steps of 2.5 min. The treated samples were plated directly or after suitable dilution onto MEA agar plates and incubated at 28 °C for 10 days to count the colony-forming units.

Viability was assessed by using non-irradiated suspensions of each strain under the same conditions as the controls. *E. coli* was used as a negative control in both gamma and UV irradiation experiments, with *D. radiodurans* R1 as a positive control.

### Maximum Tolerance Concentrations (MTC) of heavy metals

To determine the MTC of the strain for different heavy metals, Czapek medium incorporated with different concentrations of Ni^2+^, Hg^2+^, Cu^2+^, Co^2+^, Cd^2+^, Pb^2+^, and Cr^2+^ was used. The concentration range of all heavy metal ions was from 50 mg/L to 1,500 mg/L. The cells were grown under optimal culture conditions at 28 °C for 10 days^48^.

### Analytical methods

Cell growth was analyzed by measuring the optical density of the cell suspensions at 600 nm (OD_600_) on an Ultrospec 3300 pro spectrophotometer (Amersham Bioscience, United Kingdom) after appropriate dilution. Cell morphology was analyzed by direct observation under an Olympus CX23 microscope (Olympus, Japan) and a Zeiss Libra 200 transmission electron microscope (Carl Zeiss AG, Germany). The concentration of trehalose in the cell extracts was determined by the enzymatic trehalase method (Sigma-Aldrich, USA) as described previously^44^. Samples for dry weight analysis were washed with distilled water and dried at 105 °C for 12 h. TPS and ATH activities were determined as reported previously^49^. Quantitative Real-time Reverse Transcription PCR (qRT-PCR) was carried out to verify the RNA-seq results using an ABI STEPONE PLUS Real Time PCR System^50^. Primer Premier 6.0 (PREMIER Biosoft International, Palo Alto, CA, USA) was used to design primers for the target genes (Table 4). All results are based on triplicate determinations.

**Table 4:** qRT-PCR primers used in this study.

Gene	Primer	Sequence (5′ → 3′)
*rad25*	Forward	TCACAAATCCGCTTTCGAAGCCGCT
	Reserve	ATGCCGCCCAAGAGGAAAGCCCCTG
*rad1*	Forward	ATGGCCCCAACAAACACATGTCTA
	Reserve	TCACTCGTCGTCCGTCTCTGAATCA
*mutL*	Forward	ATGGAAGAGAACGGAGATGTGGAG
	Reserve	TCAACATCTTTCAAACACGCGGTAC
*svf1*	Forward	ATGATGAACTGGGCGAAACAACAGC
	Reserve	TCATGAGATGAAAGTGGCCTCAGTG
*rci1*	Forward	ATGTGCGGCAGCGATATCTTCTTGG
	Reserve	CTAGTCGTGTGACTGGACCTTGTTG
*ABC1*	Forward	CTAGATGACTGTAGGTTGCATGCCC
	Reserve	ATGAAAGGAACGTTTGGATGGGCTC
*qutD*	Forward	ATGGGTGTCCTTGCCAAAATCGAAG
	Reserve	CTACGCCCTATGCTCTGAAGACTGA
*FeSOD*	Forward	ATGGCCGTCACACAATACTCACTAC
	Reserve	TCAAATGCTCGCACGAAGGAATTTA
*ccp1*	Forward	TTACTCGGAAGGCTTGAAGGTCATG
	Reserve	ATGGCTTCCGCTGCCAGATCTTTCA
*caos*	Forward	ATGGCTCCCTCTCATCCTCTTTCCA
	Reserve	TTAGAGCTTTCCCTGAGCAGGTTG
*gsho1*	Forward	ATGTCTTCTGCTACCTCTTTCCACGA
	Reserve	TTAAGCCTTGAGCTGAGCCTCGATG

## Electronic supplementary material

Supplementary information
