# Supplementary material for: Protective role of trehalose during radiation and heavy metal stress in Aureobasidium subglaciale F134
Source: Sci Rep. 2017 Dec 14;7:17586. doi: 10.1038/s41598-017-15489-0 (PMC5730648; doi:10.1038/s41598-017-15489-0)
Supplement: Supplementary file 1 — Supplementary information [file 41598_2017_15489_MOESM1_ESM.doc]

**Protective role of trehalose during radiation and heavy metal stress in *Aureobasidium* subglaciale F134**

Tingting Liu^1^,^2^, Liying Zhu^3^, Zhiping Zhang^4^, He Huang^2^, Zhidong Zhang^5,*^, Ling Jiang^1,*^

^1^College of Food Science and Light Industry, Nanjing Tech University, Nanjing 210009, People’s Republic of China; ^2^College of Biotechnology and Pharmaceutical Engineering, Nanjing Tech University, Nanjing 210009, People’s Republic of China; ^3^College of Chemical and Molecular Engineering, Nanjing Tech University, Nanjing 210009, People’s Republic of China; ^4^ Nanjing Beishengrong Energy Technology Co. Ltd., Nanjing 210009, People’s Republic of China; ^5^Institute of Microbiology, Xinjiang Academy of Agricultural Sciences, Urumqi, Xinjiang Uigur Autonomous Region, People’s Republic of China

^*^Corresponding authors.

Ling Jiang, Email: jiangling@njtech.edu.cn, Tel: +86-25-58139942,

College of Food Science and Light Industry, Nanjing Tech University, Nanjing 210009, PR China.

Zhidong Zhang, Email: zhangzheedong@sohu.com, Tel: +86-991-4520524,

Institute of Microbiology, Xinjiang Academy of Agricultural Sciences, Urumqi, Xinjiang Uigur Autonomous Region, PR China.


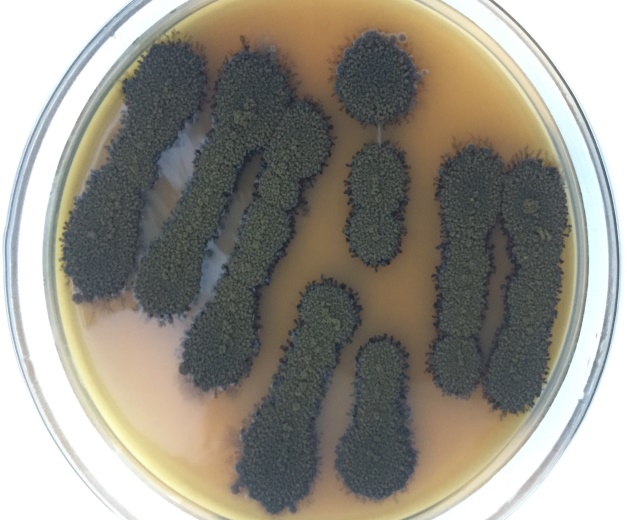

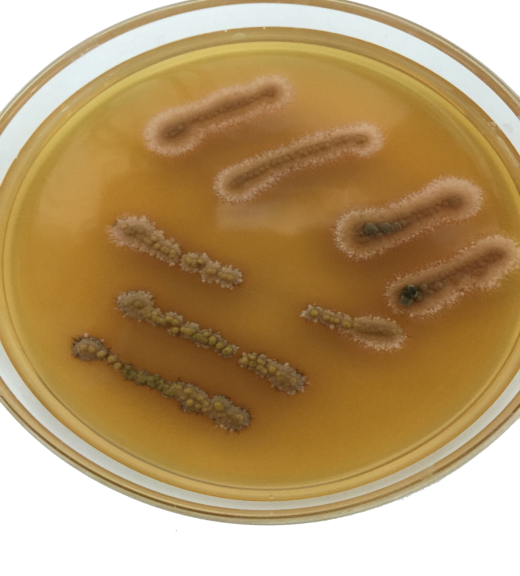

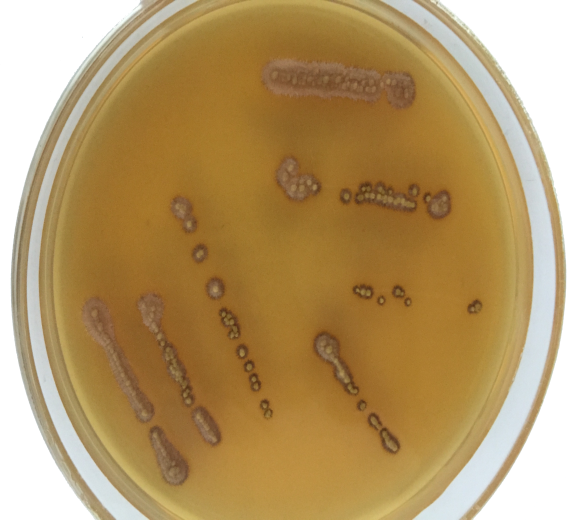


a

b

c


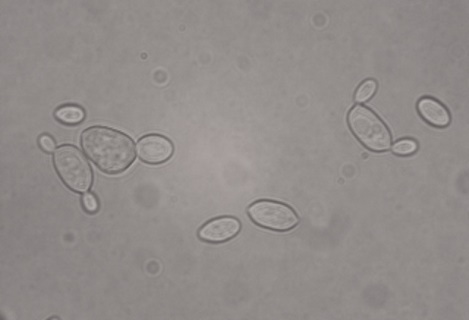

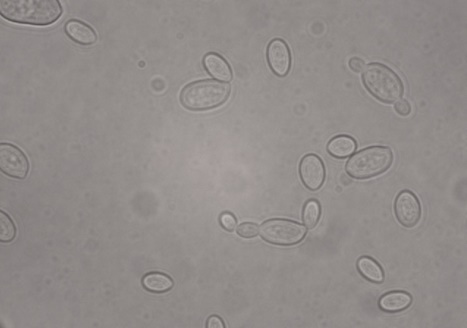

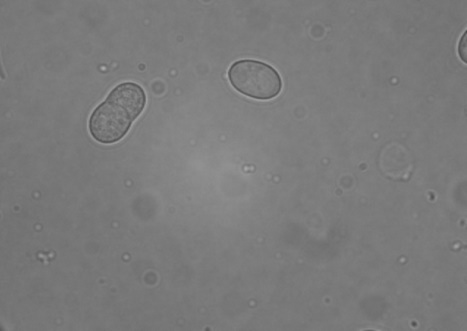

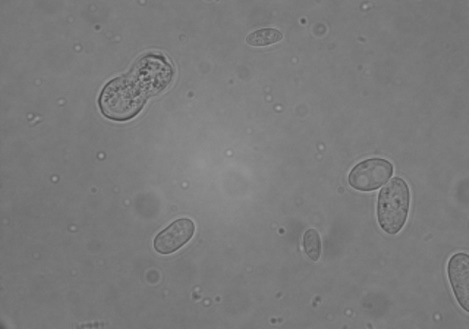


e

d

f

g

**Figure S1**. Macromorphology of *A. pullulans* sp. nov*.* F134 incubated for 3, 6, 9 days at 28 ºC on yeast extract peptone dextrose (YPD)^1^ medium (a-c). Scale bar, 10 mm. Micromorphology of different modes of reproduction: fissiparism (d-e) and budding (f-g) of *A. pullulans* sp. nov*.* F134 cultivated for 3 days at 28 ºC in YM. Scale bar, 10 μm. Initially, most colonies (4.8-8.9 mm diam.) were yeast-like, light brown, and slimy due to abundant sporulation and EPS production, with several faint yellow granular protuberances appearing on the middle surface of the colonies. After cultivation for 6 days, most colonies (9.7-13.5 mm diam.) began to turn a purplish brown, and several granular protuberances even showed dark brown color. Compared to the colony size of *A. thailandense* sp. nov.^2^, our isolated strain was still relatively small when cultured for 9 days (14.1-19.8 mm diam. vs. 24-25 mm diam.). The whole colonies presented black with white fungal mycelia embedded in the culture plates, with protuberances diminishing obviously or even disappearing. Further microscopic observation, as shown in Figure S1, revealed that the single cells varied in size from 2.9 × 3.0 μm to 7.8 × 8.2 μm, which was smaller than what was reported for the type strain of *A. pullulans*^3^.


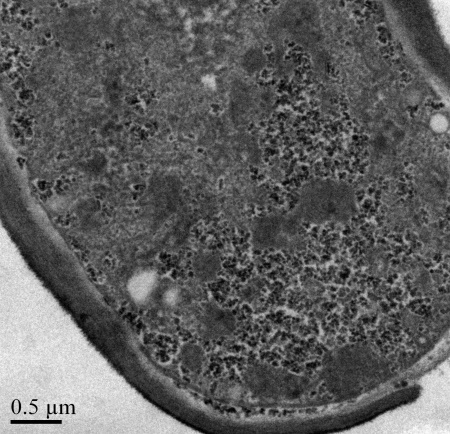

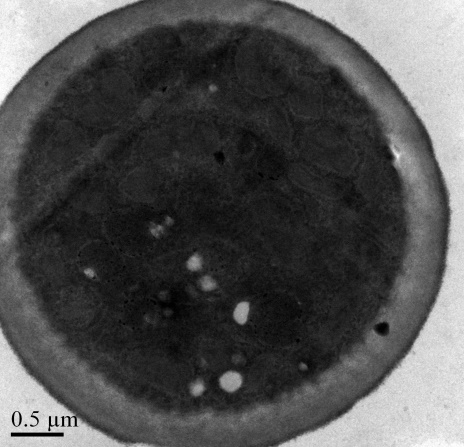


**Figure S2**. TEM of *A. pullulans sp.* nov*.* F134 incubated in YPD medium at 28 ºC for 7 days. The capsules of the chlamydospores appeared slightly granular and lumpy, with a cytoderm thickness of 0.3-0.5 μm.

**Table S1.** Comparison of carbon and nitrogen assimilation profile among *A.* subglaciale F134, *A.* subglaciale NRRL Y-7469, and *A.* subglaciale NRRL Y-2311-1

| **Carbon and nitrogen sources** | **F134** | **NRRL Y-7469^a^** | **NRRL Y-2311-1^a^** |
| --- | --- | --- | --- |
| D-glucose | + | + | + |
| D-xylose | + | + | + |
| D-fructose | w | + | + |
| L-rhamnose | + | + | + |
| D-maltose | + | + | + |
| D-sucrose | + | + | + |
| D-raffinose | + | + | + |
| D-trehalose | + | + | + |
| D-cellobiose | + | + | + |
| D-xylitol | + | + | + |
| D-sorbitol | + | + | + |
| sodium citrate | – | + | + |
| sodium acetate | – | w | + |
| sodium nitrite | + | + | + |
| sodium nitrate | + | + | + |
| L-arginine | + | + | + |
| L-threonine | – | – | – |
| L-asparagine | – | + | + |
| glycine | w | + | + |
| L-proline | + | + | + |
| L-glutamine | – | + | + |
| L-alanine | + | + | + |
| L-serine | + | + | + |

+: growth; w: weak growth; –: no growth.

^a^*A. pullulans* NRRL Y-7469 and *A. pullulans* NRRL Y-2311-1 are both standard strains.

The obtained isolates were first investigated regarding their assimilation of different carbon and nitrogen sources, and the growth experiments were conducted at least twice^4^. Inocula were cultivated in yeast extract-malt extract (YM)^5^ medium (yeast extract 0.3%, glucose 1%, malt extract 0.3%, peptone 0.5%) at 28 °C for 7 days. A corresponding amount of sterile water was used instead of the inoculum as a control. The colony diameter was considered as reference standard: positive, weak and negative^6^. The carbon sources assimilated by isolate F134 encompassed common monosaccharides, oligosaccharides, and saccharide derivatives, but the strain grew weakly on D-fructose and no growth was observed on sodium citrate and sodium acetate, which was different to both *A. pullulans* NRRL Y-7469 and NRRL Y-2311-1^7^. The nitrogen sources assimilated by isolate F134 included L-arginine, L-proline, L-alanine and L-serine, which has also been reported for a large number of other *A. pullulans* strains^8^. However, L-threonine, L-asparagine and L-glutamine were not assimilated by *A*. subglaciale F134, which was different from another type strain *A. pullulans* FB-1^9^.

**References**

1. Schmitt, M. E., Brown, T. A. & Trumpower, B. L. A rapid and simple method for preparation of RNA from *Saccharomyces cerevisiae*. *Nucleic. Acids Res*. **18**, 3091-3092 (1990).
2. Arzanlou, M. & Khodaei, S. *Aureobasidium iranianum*, a new species on bamboo from Iran. *Mycosphere* **3(4)**, 404-408 (2012).
3. Punnapayak, H., Sudhadham, M., Prasongsuk, S. & Pichayangkura, S. Characterization of *Aureobasidium pullulans* isolated from airborne spores in Thailand. *J. Ind. Microbiol. Biot.* **30**, 89-94 (2003).
4. Ferreira, A. D. & Viljoen, B. C. Yeasts as adjunct starters in matured Cheddar cheese. *Int. J. Food Microbiol.* **86**, 131-140 (2003).
5. Psani, M. & Kotzekidou, P. Technological characteristics of yeast strains and their potential as starter adjuncts in Greek-style black olive fermentation. *World J. Microb. Biot*. **22**, 1329-1336 (2006).
6. Kane, J. & Summerbell, R. C. Sodium chloride as aid in identification of *Phaeoannellomyces werneckii* and other medically important dematiaceous fungi. *J. Clin. Microbiol.* **25**, 944-946 (1987).
7. Prasongsuk, S., Sullivan, R., Kuhirun, M., Eveleigh, D. & Punnapayak, H. Thailand habitats as sources of pullulan-producing strains of *Aureobasidium pullulans*. *World J. Microb. Biot.* **21**, 393-398 (2005).
8. De, H. G. & Yurlova, N. Conidiogenesis, nutritional physiology and taxonomy of *Aureobasidium* and *Hormonema*. *Anton. Leeuw*. *Int. J. G.* **65**, 41-54 (1994).
9. Singh, R. & Saini, G. Pullulan-hyperproducing color variant strain of *Aureobasidium pullulans* FB-1 newly isolated from phylloplane of *Ficus* sp. *Bioresource Technol.* **99**, 3896-3899 (2008).
